# Evaluating health workers attitude towards implementation of maternal and perinatal deaths surveillance and response system in Morogoro region; analytical cross-sectional study

**DOI:** 10.1371/journal.pone.0300665

**Published:** 2024-04-01

**Authors:** Christina Kashililika, Rehema Bakari, Fabiola Moshi

**Affiliations:** 1 Department of Clinical Nursing, School of Nursing and Public Health of the University of Dodoma, Dodoma, Tanzania; 2 Department of Nursing Management and Education, School of Nursing and Public Health of the University of Dodoma, Dodoma, Tanzania; Public Library of Science, UNITED KINGDOM

## Abstract

Maternal and Perinatal Deaths Review and Surveillance (MPDSR) is a technical system which was issued by the World Health Organization in 2013 to help developing countries improve maternal health. The major purpose of the system was to reduce the ongoing high numbers of maternal deaths and perinatal deaths from avertable causes. Tanzania adopted MPDSR system in 2015. The study aimed to assess health workers attitude towards implementation of MPDSR system in Morogoro Region. This analytical cross-sectional study was conducted in three districts of Morogoro region from April 27, 2020 to May 29, 2020 involving 360 health workers from 38 health facilities. A semi-structured questionnaire was used for data collection. SPSS software version 25 was used to analyze the obtained data. Descriptive analysis was done to describe the characteristics of study participants. Binary logistic regression analysis was used to assess predictors of health workers attitude towards the MPDSR system. A total of 255(70.8%) of respondents had positive attitude towards MPDSR system. After controlling of confounders predictor of positive attitude were location of health facility [rural (AOR = 0.216 at 95% CI = 0.121–0.387, p = <0.001)], Age group [Below 30(AOR = 0.459 at 95%CI = 0.264–0.796, p = 0.006)] and status of training on MPDSR [Yes (AOR = 4.892 at 95%CI = 2.187–10.942, P = <0.001)]. Substantial number of health workers had positive attitude towards the MPDSR system. Health workers who were residing in rural settings and younger than 30 years were less likely to have positive attitude towards the system. Health workers who had access to be trained about the system were more likely to have positive attitude towards MPDSR system. The study recommends the training of health workers about the system so as to increase their attitude and hence the use of the system.

## Introduction

Health workers and stakeholders play a vital role in promoting community health while working harder in avoiding the occurrence of preventable maternal and perinatal deaths. In 2017 it was estimated that during pregnancy and childbirth period, 295000 women died worldwide while most of these deaths occur in developing and poor resourced countries [1, 2]. In response to this tremendously increase of maternal deaths, WHO and UNPFA developed an integrated disease surveillance and riposte guideline and disseminate to all countries and in 2018 included perinatal death in the revised guideline [1, 2]. While maternal and perinatal deaths continue to be a problem globally, the reports show that the countries with highest mortality burden poorly records perinatal deaths [3, 4].

Furthermore, WHO introduced a globally comparable and applicable system as a first step in addressing maternal and perinatal mortality through capturing and classifying the causes of those deaths accurately worldwide [2]. MPDSR as global system introduced is a key initiative in improving the quality of care for maternal health, involves qualitative and in-depth inquiry of the causes and circumstances encircling maternal and perinatal deaths [2]. It is suggested that 30–35% maternal and perinatal mortality reduction can be observed if maternal and perinatal death reviews are completed while implementations of recommendations are done alongside pedagogical development of local supervision [1, 2].

However, as it help to address quality of care deficits, several studies have shown challenges towards implementation of MPDSR such as lack of MPDSR policy, lack of diagnostic capacity to accurately classify the causes of the deaths and gaps in pinpointing and recording maternal and perinatal deaths [2]. Thus, if the main system that is supposed to record maternal and perinatal deaths is not fully operating then there is great risk that other maternal and perinatal deaths are not recorded [1, 2]. In sub–Saharan Africa, over 40 percent of preventable maternal and perinatal deaths occurs while perinatal deaths accounts for about one half [3]. The studies from eastern and southern Africa reveled challenges like legal framework, lack of knowledge and accountability hinder the implementation of MPDSR system [5–7].

In Tanzania, maternal death reviews have been conducted since 2015 when the system was introduced to rule out the causes and risk factors contributing to maternal deaths through collection of information medical records, health care providers and relatives’ interviews [8]. The information obtained are used for discussion during MPDSR meetings for suggestions of action points to stir up action at local and national health facilities. However, the health facilities face various challenges in documentation and record keeping of medical files which results into insufficiency of the information discussed during meetings [9].

A study conducted in northern Tanzania showed fragmentation of implementation of the system due to separation of work between MPDSR and quality improvement teams [6]. On the other study done in Mtwara region showed that health providers never received formal training while reginal and district health providers received and also there were poor dissemination of the guideline [10]. Other finding was that some of health providers lacked commitment and had poor attitude towards implementation of MPDSR which demanded continuous supervision [8].

Moreover, apart from the extrinsic factors outlined by different studies, the intrinsic factors such as self-efficacy, individual motivation and identification with the intervention plays a great role in implementation of MPDSR [3]. The health care provider with positive attitude will be self-motivated and confidently able to implement MPDSR as they are willingly to do so. On the other side, poor attitude will lead to poor implementation and finally loss of crucial MPDSR information. It is therefore important to investigate the attitude of healthcare providers towards implementation of MPDSR system as through them is where maternal and perinatal information are obtained.

## Materials and methods

### Study design

A hospital-based analytical cross-sectional study employing a quantitative approach was used. The study population were health workers who are responsible to participate in MPDSR system.

### Study area

It was conducted in three districts of Morogoro region which were Morogoro municipal, Mvomero district and Kilosa district. The region is one of 31 regions of Tanzania located in the country’s eastern zone. It has a population of 2,218,492 and is made up of seven districts and nine councils. The region has 375 health facilities and is among the top five regions with highest number of health facilities in Tanzania. During the time of the study Morogoro region had a of total number of 2846 skilled health workers employed by government. The region was chosen to be location of the study because of its high maternal and perinatal mortality rate and the fact that no study related to MPDSR had been done in the region before [11].

### Inclusion criteria

The inclusion criteria for this study was all health care workers who were in service for at least one year prior to data collection period.

### Exclusion criteria

All health workers who were working on the health facility for part-time basis were excluded to participate in this study.

### Sample size estimation

A number of participating health facilities (n_1_) and health workers (n_2_) was calculated from the formula for cross sectional study for finite population [11] which is shown in Eq 1.

$$\text{n}=\frac{\frac{{\text{t}}^{2}\text{P}(1-\text{P})}{{\text{e}}^{2}}}{1+\frac{1}{\text{N}}\left(\frac{{\text{t}}^{2}\text{P}(1-\text{P})-1}{{\text{e}}^{2}}\right)}$$ (1)

Where, N = the size of the target population

t = the critical value for the given confidence level, in this study the confidence level was 95%, therefore t was equal to Z_0.05_ which is 1.96

e = the margin error, taken for 0.05

p = the proportion of health workers who had adequate knowledge on MPDSR system from previous studies which is 60.6% [12].

From above formula, the sample size for health workers ‘n_1_’ was given as follows:

$${\text{n}}_{1}=\frac{\frac{{\left(1.96\right)}^{2}\times \text{0}.606(1-0.606)}{{\left(0.05\right)}^{2}}}{1+\frac{1}{\text{2846}}\left(\frac{{\left(1.96\right)}^{2}\times \text{0}.606(1-0.606)}{{\left(0.05\right)}^{2}}-1\right)}$$

$${\text{n}}_{1}=360$$

Adding attrition rate of 10% gives a total sample size of 360 health care workers from Morogoro region.

### Sampling technique

The study used a multistage sampling technique for selection of study participants. In the first stage, three districts with highest number of health facilities were purposively selected. The selected facilities were Morogoro MC, Mvomero DC and Kilosa DC. In the second stage, a stratification sampling technique was used to select 10 facilities in Morogoro Municipal Council, 15 facilities in Mvomero District Council and 13 facilities in Kilosa District Council. In the third stage, each council, hospitals were purposively selected because of high number of health workers, their relatively higher volumes of deliveries and the fact that hospitals manage most of complications of pregnancy and child birth, while health centers were randomly selected.

In the fourth stage, a simple random sampling by lottery replacement was used to select health workers. In each council facilities contributed equal number of health workers; 12 from each facility in Morogoro Municipal Council, 8 from each facility in Mvomero District Council and 9 or 10 from each facility in Kilosa District Council [11].

### Data collection technique and tool

A semi-structured questionnaire developed using the MPDSR system guide was used to collect data about knowledge on MPDSR system among health workers in Morogoro Region. This analytical cross-sectional study was conducted in three districts of Morogoro region from April 27, 2020 to May 29, 2020.

### Variable measurements

Attitude towards MPDSR among health workers was defined in this study as the extent to which health workers perceive MPDSR system. Attitude towards MPDSR system was measured by using a questionnaire that was designed in 5-point Likert scale with 10 Likert items, each with five levels of agreement; the distribution of points was 5 points for strongly agree choice to 1 point for strongly disagree choice. A participant was asked to read each of the 10 statements and select his or her most appropriate level of agreement for the statement by putting a tick in the respective box. A total score was 50 points. A score above 30 points was termed as positive attitude while a score of 30 or below was considered as negative attitude.

### Data analysis procedure

The obtained quantitative data was analyzed using SPSS software for both descriptive statistics (frequency distribution and Chi-square) and inferential statistics (Binary logistic regression analysis) to predict factors associated attitude towards MPDSR system among health workers in Morogoro.

### Ethical approval

The approval for conducting this study was obtained from the University of Dodoma Research and Ethical Clearance Committee with. The permission to collect data was obtained from Morogoro Municipal Director as well as from Medical officer in-charge of the three districts. Both verbal and written informed consent was obtained from the study participants prior to conducting a study and those who consented were included in the study. Confidentiality and anonymity were well adhered through the use of code numbers on questionnaire rather than actual names.

## Results

### Socio-demographic characteristics

A total of 360 health workers from hospitals and health centers participated in the study with a response rate of 100%. Study findings revealed that 69.7% (n = 251) were respondents aging 30 years or above and 69.8% (n = 244) were females. About 52.5% (n = 189) of the respondents had education of diploma level while those with lowest and highest education level were certificates holders 40.6% (n = 146) and bachelor holders 2.5% (n = 9) respectively. On the other hand, nurses were 54.2% (n = 195). Respondents who were trained on MPDSR were 21.4% (n = 77) “Table 1”.

**Table 1:** Demographic characteristics of the study respondents (N = 360).

Variables	Frequency (n)	Percentage (%)
Age group		
200Below 30	109	30.3
30 or above	251	69.7
Sex		
Male	116	32.2
Female	244	67.8
Education		
Certificate	146	40.6
Diploma	189	52.5
Advanced Diploma	16	4.4
Bachelor	9	2.5
Profession		
Nursing	195	54.2
Clinician	63	17.5
Others	102	27.3
Duty station		
Antenatal ward	66	18.3
Labor ward	101	28.1
Postnatal ward	69	19.2
OPD	70	19.4
Others	54	15.0
Duration in practice		
1–5 years	136	37.8
6–10 years	128	35.6
Above 10 years	96	26.7
Training on MPDSR system		
Yes	77	21.4
No	283	78.6

### Attitude towards MPDSR system

#### Item distribution of attitude of health workers on MPDSR program

“Table 2” shows items that were used to measure knowledge and the distribution of score for each item.

**Table 2:** Item distribution of attitude of health workers on MPDSR program.

Item	Strongly disagree n (%)	Disagree n (%)	Neutral n (%)	Agree n (%)	Strongly Agree n (%)
Conducting maternal and perinatal death reviews could improve maternal and perinatal care.	36 (10.0)	45 (12.5)	15 (4.2)	141 (39.2)	123 (34.2)
Implementing MPDSR increases work load to health workers	43 (11.9)	76 (21.1)	40 (11.1)	127 (35.3)	74 (20.6)
Sometimes MPDSR meetings are inconvenient to be because are too long.	54 (15.0)	86 (23.9)	35 (9.7)	126 (35.0)	59 (16.4)
Many of the recommendations we set during meetings are useful elsewhere practice	35 (9.7)	65 (18.1)	21 (5.8)	138 (38.3)	101 (28.1)
Because of MPDSR it is stressful to work in departments/units that involve maternal and neonatal care.	40 (11.1)	84 (23.3)	38 (10.6)	125 (34.7)	73 (20.3)
MPDSR affects how health workers provide maternal and newborn care	80 (22.2)	120 (33.3)	26 (7.2)	92 (25.6)	42 (11.7)
My capacity to conduct MPDSR was built by district or ministry of health.	48 (13.3)	106 (29.4)	30 (8.3)	114 (31.7)	62 (17.2)
I feel encouraged to conduct MPDSR	36 (10.0)	59 (16.4)	29 (8.1)	155 (43.1)	81 (22.5)
I like to know the implementation status of every recommendation that are set out during meetings	46 (12.8)	53 (14.7)	31 (8.6)	147 (40.8)	83 (23.1)
Every health care worker will need to know about MPDSR in their carrier.	49 (13.6)	59 (16.4)	31 (8.6)	116 (32.2)	105 (29.2)

#### Health workers attitude towards MPDSR system

**Fig 1:**
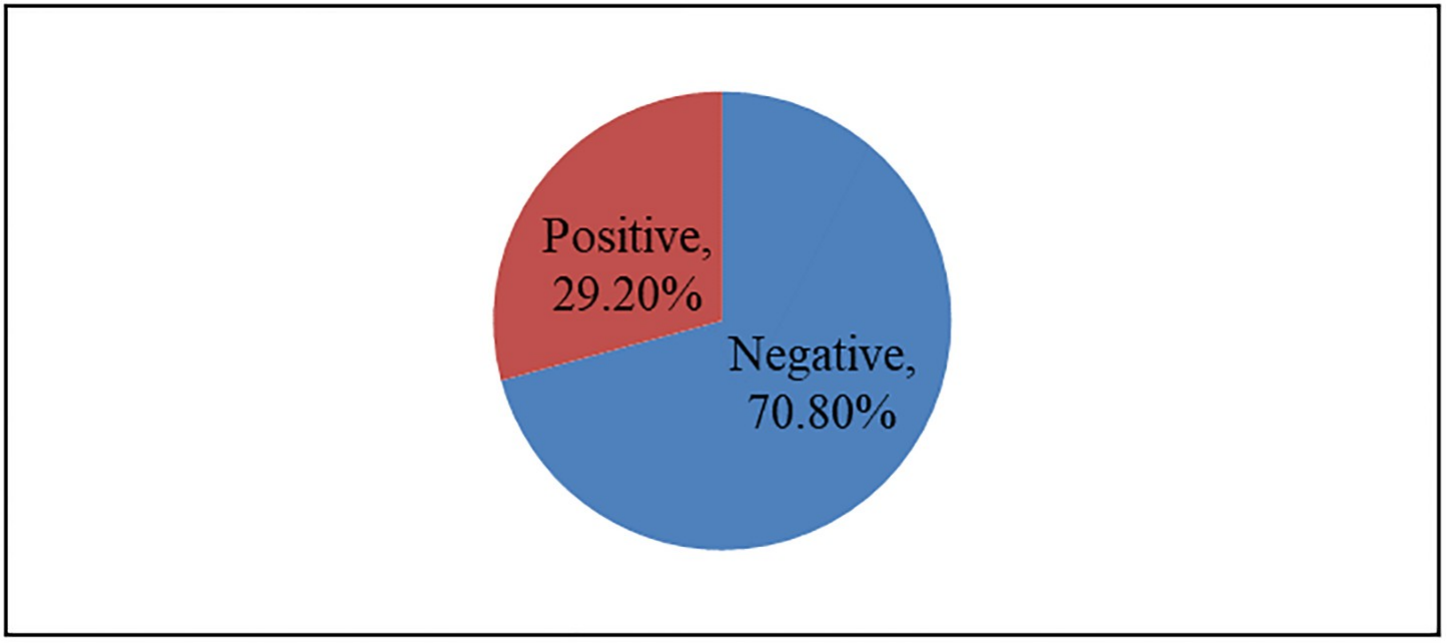
Attitude of health workers towards MPDSR program.

Out of all respondents (N = 360), about 70.8% (n = 255) had positive attitude towards MPDSR system while 29.2% (n = 105) had negative attitude towards the system. See “Fig 1”.

#### MPDSR training status among health workers in Morogoro region

Majority of health workers who were trained on MPDSR were those Morogoro Municipal Council, from urban health facilities, female health workers, nursing health workers and those who aged above 30 years “Table 3”.

**Table 3:** MPDSR training status among health workers in Morogoro region.

Variables	Trained on MPDSR	Not trained on MPDSR
**Districts**		
Morogoro MC	41(33.9)	80(66.1)
Mvomero DC	20(16.8)	99(83.2)
Kilosa DC	16(13.3)	104(86.7)
**Location of health facility**		
Urban	41(33.9)	80(66.1)
Rural	36(15.1)	203(84.9)
**Sex of the participant**		
Male	20(17.2)	96(82.8)
Female	57(23.4)	96(76.6)
**Level of education of participant**		
Certificate	40(27.4)	106(72.6)
Diploma	30(15.9)	159(84.1)
Advanced diploma	5(31.3)	11(68.8)
Bachelor	2(22.2)	7(77.8)
**Profession of participant**		
Nursing	51(26.2)	144(73.8)
Clinician	11(17.5)	52(82.5)
Others	15(14.7)	87(85.3)
Duty station		
antenatal ward	15(22.7)	51(77.3)
Labor ward	30(29.7)	71(70.3)
Postnatal ward	11(15.9)	58(84.1)
OPD	15(21.4)	55(78.6)
Others	6(11.1)	48(88.9)
Age_groups		
Below 30	11(10.1)	98(89.9)
30 or above	66(26.3)	185(73.7)

#### The relationship between health workers characteristics and attitude towards MPDSR program

Variables which showed significant relationship with attitude of health workers on MPDSR were location of health facility (X^2^ = 13.04, p = <0.001), age group of respondents (X^2^ = 8.001, p = 0.005), education level (X^2^ = 8.663, p = 0.034), profession of respondent (X^2^ = 7.363, p = 0.026), duty station of respondent (X^2^ = 10.51, p = 0.033) and health worker’s status of training on MPDSR (X^2^ = 14.48, p = <0.001). See “Table 4”.

**Table 4:** The relationship between health workers characteristics and attitude towards MPDSR program.

Variable	Positive attitude n (%)	Negative attitude n (%)	X^2^	p-value
**Characteristic of health facility**				
Hospital	132 (72.5)	50 (27.5)		
Health center	123 (69.1)	55 (30.9)	0.511	0.475
**Location**				
Urban	71 (58.7)	50 (41.3)		
Rural	184 (77.0)	55 (23.0)	13.04	<0.001
**Age group**				
Below 30	66 (60.6)	43 (39.4)		
30 and above	189 (75.3)	62 (24.7)	8.001	0.005
**Sex**				
Male	77 (66.4)	39 (33.6)		
Female	178 (73.0)	66 (27.0)	1.643	0.200
**Level of education**				
Certificate	103 (70.5)	43 (29.5)		
Diploma	128 (67.7)	61 (32.3)		
Advanced diploma	15 (93.8)	1 (6.2)		
Bachelor	9 (100.0)	0 (0.0)	8.663	0.034
**Profession**				
Nurse	144 (73.8)	51 (26.2)		
Clinician	49(77.8)	14 (22.2)		
Others	62(60.8)	40 (39.2)	7.313	0.026
**Duty station**				
Antenatal ward	49 (74.2)	17(25.8)		
Labor ward	76 (75.2)	25 (24.8)		
Postnatal ward	45 (65.2)	24 (34.8)		
OPD	55 (78.6)	15 (21.4)		
Other units	30(55.6)	24 (44.4)	10.51	0.033
**Training on MPDSR**				
Yes	68 (88.3)	9 (11.7)		
No	187 (66.1)	96 (33.9)	14.48	<0.001

#### Association between health workers characteristics and attitude towards MPDSR program

After controlling of confounders predictor of attitude were location of health facility [rural (AOR = 0.216 at 95% CI = 0.121–0.387, p = <0.001)], Age group [Below 30(AOR = 0.459 at 95%CI = 0.264–0.796, p = 0.006)] and status of training on MPDSR [Yes (AOR = 4.892 at 95%CI = 2.187–10.942, P = <0.001)] as shown in “Table 5”.

**Table 5:** Association between health workers characteristics and attitude towards MPDSR program.

Variable	OR	95% CI		P-value	AOR	95% CI		P-value
		Lower	Upper			Lower	Upper	
**Location**								
Urban	1				1			
Rural	0.424	0.265	0.680	<0.001	0.216	0.121	0.387	<0.001
**Age**								
30 or above	1				1			
Below 30	0.504	0.312	0.813	0.005	0.459	0.264	0.796	0.006
**Education**								
Bachelor	1				1			
Advanced diploma	1.142	0.714	1.824	0.058	1.096	0.622	1.934	0.750
Diploma	0.160	0.020	1.247	0.080	0.228	0.026	2.037	0.186
Certificate	0.000	0.000	-	0.999	<0.001	0.000		0.999
**Profession**								
Nurse	1				1			
Clinician	0.807	0.411	1.583	0.532	1.104	0.361	3.374	0.863
Others	1.822	1.094	3.033	0.021	1.029	0.380	2.790	0.955
**Station**								
Antenatal ward	1				1			
Labor ward	0.948	0.465	1.935	0.884	0.923	0.420	2.027	0.716
Postnatal ward	1.537	0.732	3.227	0.256	1.536	0.568	4.154	0.274
OPD	0.786	0.355	1.739	0.552	0.577	0.176	1.893	0.305
Other units	2.306	1.068	4.979	0.033	1.973	0.563	6.916	0.184
**Training**								
No	1				1			
Yes	<0.001	3.879	1.855	8.109	4.892	2.187	10.94	<0.001

## Discussion

This study found that majority of health workers had positive attitude towards MPDSR system. Similar findings were obtained from a study done in Dar es Salaam by Nyamtema, Urassa, Pembe, Kisanga, and Van Roosmalen in 2010 [14] who demonstrated high level of positive attitude among health workers affected efficient audit system. Furthermore, it was found in this study that having health care providers with positive attitude in a facility is about nine times more likely to have the health facility with satisfactory level of MPDSR implementation.

In this study it was also found that facilities whose staff have positive attitude towards MPDSR are about nine times more likely to have satisfactory level of MPDSR implementation than their counterpart. This finding is supported by results done in Uganda by Agaro, et al. in 2016 [13] who concluded that there are different health workers factors that influence MPDSR implementation status. Although majority of participants had positive attitude and were encouraged and agreed that conducting MPDSR would improve maternal and perinatal care they also agreed that MPDSR implementation increases their workload and at the same time they are stressed to work in the units involving maternal and neonatal care thereby affecting provision of care as shown in different studies [7, 14].

Moreover, factors such as location of the health center, health care workers’ level of education, duty station and training of the MPDSR program contribute to the attitude of the health care workers towards implementing of the MPDSR. Thus, training health care workers was found to have strong positive association with their attitude. This finding supports the need to involve and train health care providers on MPDSR system which has positive effect on MPDSR implementation. On point of discussion, conducive and motivating environment such as provision of incentives to health care workers implementing MPDSR could boost up their attitudes hence improve provision of care.

## Conclusion

Substantial number of health workers had positive attitude towards the MPDSR system. Health workers who were residing in urban settings and younger than 30 years were less likely to have positive attitude towards the system. Health workers who had access to be trained about the system were more likely to have positive attitude towards MPDSR system. The study recommends the training of health workers about the system so as to increase their attitude and hence the use of the system.

## Supporting information

S1 Data A dataset regarding health workers attitude towards implementation of MPDSR system at Morogoro region. (XLSX)
